# Reconstruction of the Medial Collateral Ligament Complex With a Flat Allograft Semitendinosus Tendon

**DOI:** 10.1016/j.eats.2023.09.012

**Published:** 2023-12-25

**Authors:** Wolf Petersen, Hassan Al Mustafa, Johannes Buitenhuis, Martin Häner, Karl Braun

**Affiliations:** Department for Orthopaedics and Trauma Surgery, Martin Luther Hospital, Berlin, Germany

## Abstract

The aim of this Technical Note is to reconstruct the medial collateral ligament complex with the superior medial collateral ligament and posterior oblique ligament as anatomically as possible. An allograft or contralateral semitendinosus autograft is used for anatomic reconstruction of the superior medial collateral ligament and posterior oblique ligament. After bony fixation, the tendon bundles are sutured to the remnants of the medial collateral ligament complex. Thus, the tubular grafts are pulled apart to form a flat shape that resembles that of the normal medial ligaments.

The medial collateral ligament complex is an important passive stabilizer of the knee joint and primarily stabilizes the knee against valgus stress.^1^, ^2^, ^3^ However, the medial ligamentous structures also provide secondary stabilization against posterior and anterior tibial translation.^1^, ^2^, ^3^ Anatomically, the medial collateral ligament complex consists of the superior medial collateral ligament (sMCL), deep medial collateral ligament, posterior oblique ligament (POL), and posterior medial capsule (Fig 1).^2^ The different parts of the medial collateral ligament complex have different functions stabilizing the joint against valgus stress as well as against anterior and posterior translation.^4^

**Fig 1:**
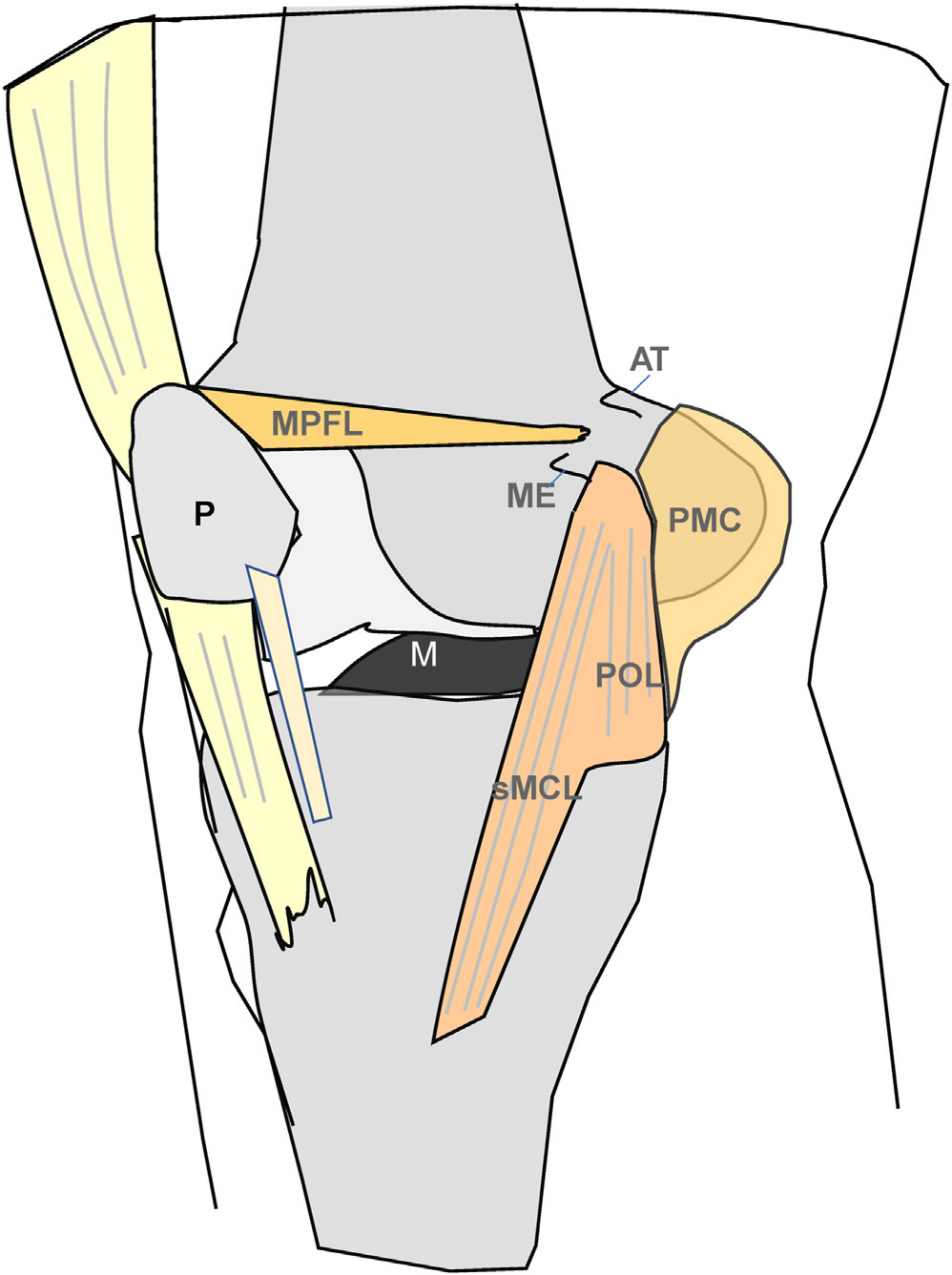
Anatomic drawing of a right knee. The medial collateral ligament complex consists of the superior medial collateral ligament (sMCL), deep medial collateral ligament, posterior oblique ligament (POL), and posterior medial capsule (PMC). (AT, adductor tubercle; M, meniscus; ME, medial epicondyle; MPFL, medial patellofemoral ligament; P, patella.)

In third-degree chronic instability, if there is insufficient residual tissue for suture or augmentation, reconstruction of the medial collateral ligament complex with a tendon graft is recommended.^5^^,^^6^ In most reconstruction techniques, the sMCL and POL are reconstructed with a tubular semitendinosus tendon graft consisting of 2 bundles.^7^, ^8^, ^9^ However, these surgical techniques do not take into account that the structures of the medial collateral ligament complex do not have a tubular shape but a flat one.^7^

Over the past years, our working group has also further developed our medial reconstruction technique,^9^ which takes the flat shape of the structure of the posteromedial complex into account. The aim of this article is to present this surgical method.

## Surgical Technique (With Video Illustration)

**Fig 2:**
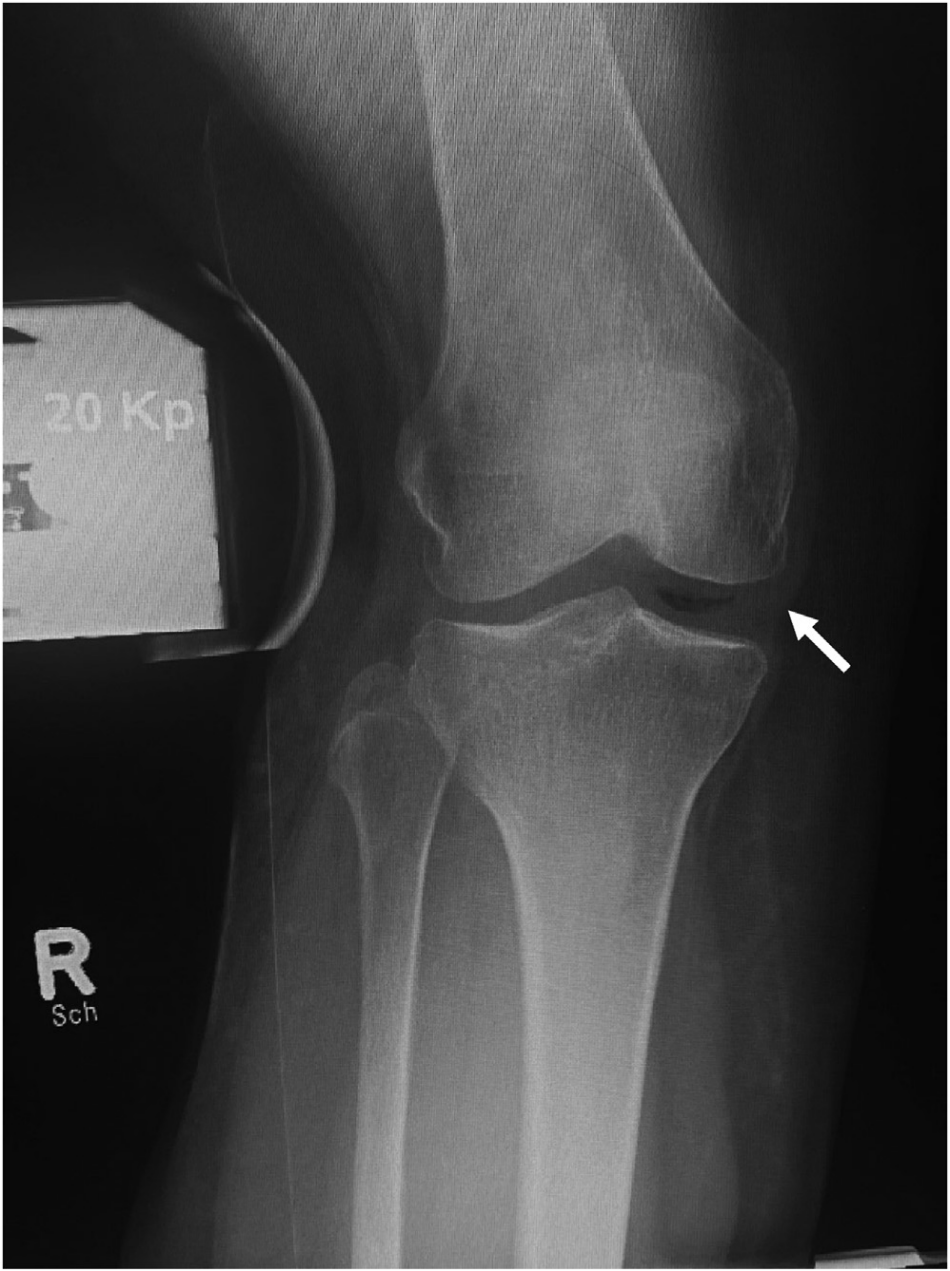
Typical valgus stress radiograph with a medial joint opening of 12 mm.

Indications for reconstruction of the posteromedial structures are listed in Table 1. Table 2 shows the preoperative diagnostic measures, and the surgical instruments needed are listed in Table 3. Figure 2 shows a typical valgus stress radiograph with a medial joint opening of 12 mm. Video 1 shows the technique described to follow.

**Table 1:** Indications for Medial Collateral Ligament Reconstruction

Indications
Symptomatic chronic isolated medial instability (valgus stress test ++ or +++ in extension without endpoint) Symptomatic chronic high-grade combined medial instability with injury of the anterior and/or posterior cruciate ligament

**Table 2:** Preoperative Diagnostics

Diagnostics
Clinical examination with Lachman test, pivot shift, valgus and varus stress in 0° and 20°, posterior drawer, dial test Magnetic resonance imaging Valgus stress radiographs (ipsi- and contralateral) Posterior stress radiographs to determine the amount of associated posterior instability Long leg radiographs to determine the leg axis

**Table 3:** Equipment Required for Medial Collateral Ligament Reconstruction

Special Instrumentation and Implants
Special motorized leg holder (not absolutely necessary) High-strength suture threads (eg, Arthrex FiberWire No. 5 for reinforcement of the tendon ends) Button for cortical fixations (eg, adjustable loops or continuous loops) Slowly absorbing suture material Eyelet wire Cannulated drills with diameters from 4.5 to 7 mm Fluoroscope

### Patient Positioning

The operation is performed with the patient in the supine position and begins with an examination of the knee joint stability under anaesthesia (Lachman, pivot shift, drawer test, dial test, varus and valgus stress in full extension and 20°). Then a tourniquet is applied, and the injured leg is draped in the usual sterile manner. Placing the leg in a movable, motorized leg holder facilitates the operation (Maquet GmbH, Rastatt, Germany).

### Diagnostic Arthroscopy

**Fig 3:**
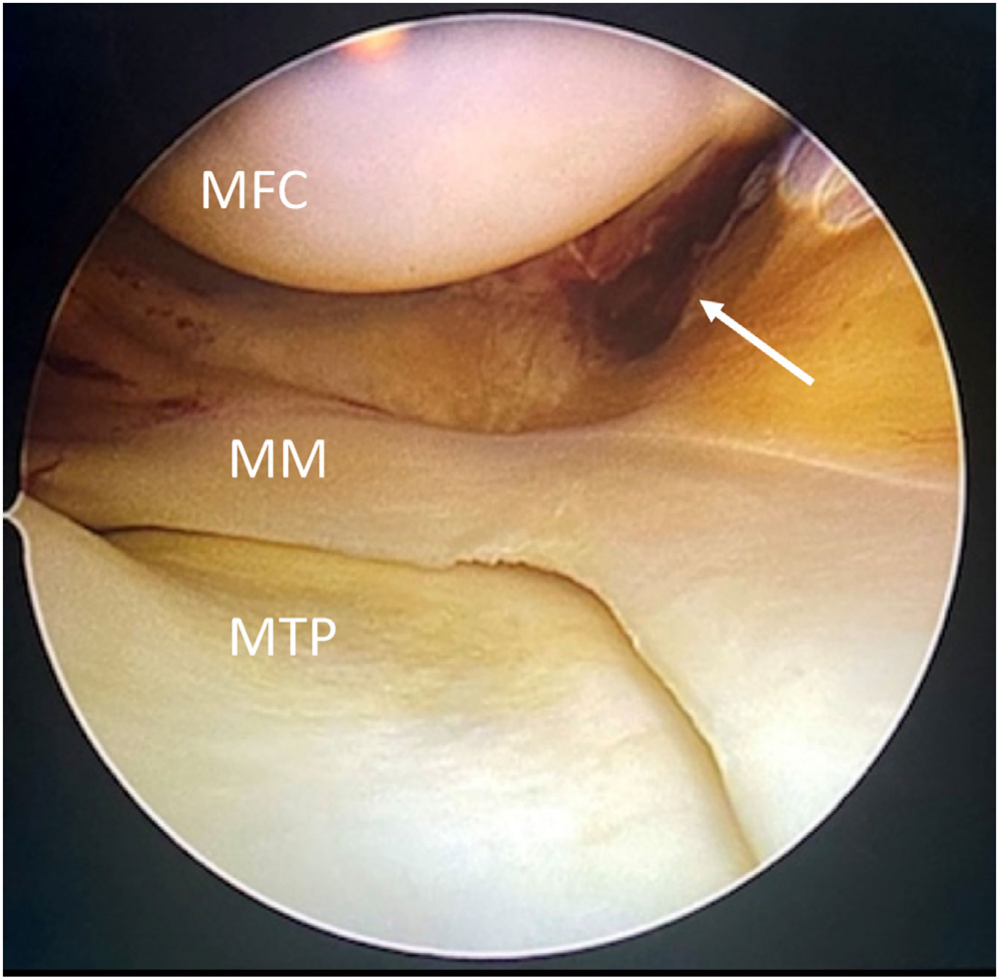
Arthroscopic view into the medial compartment (right knee) showing the “drive-through” sign. (MFC, medial femoral condyle; MN, medial meniscus; MTP, medial tibial plateau.)

A diagnostic arthroscopy using standard anterolateral and anteromedial portals is performed to diagnose and treat accompanying intra-articular injury. Typically, the “drive-through” sign can be seen in the case of high-grade medial instability (Fig 3). The position of the meniscus can provide useful information about the location of the medial ligament damage to decide where to place an additional plication of the residual medial ligamentous tissue. In the case of associated anterior or posterior instability, simultaneous arthroscopic anterior cruciate ligament or posterior cruciate ligament reconstruction can be performed.^4^^,^^10^

### Open Reconstruction of the Medial Collateral Ligament Complex

**Fig 4:**
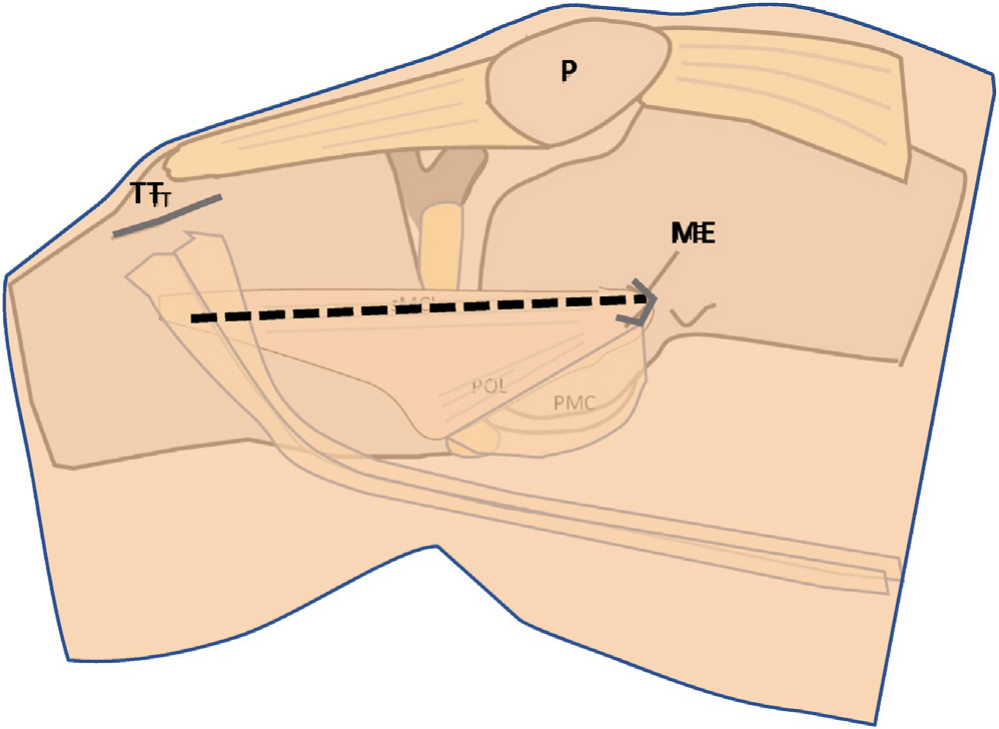
Skin incision from the medial epicondyle to the level of the tibial tuberosity. Right knee of a patient in supine position. (ME, medial epicondyle; P, patella; TT, tibial tuberosity.)

If no allograft is available or if the patient decides against the use of an allograft, a semitendinosus tendon must be harvested on the contralateral side. The harvesting technique has been described previously.^11^^,^^12^ For this medial reconstruction technique, an approximately 10- to 15-cm-long skin incision from the medial epicondyle to the level of the tibial tuberosity is needed (Fig 4).

**Fig 5:**
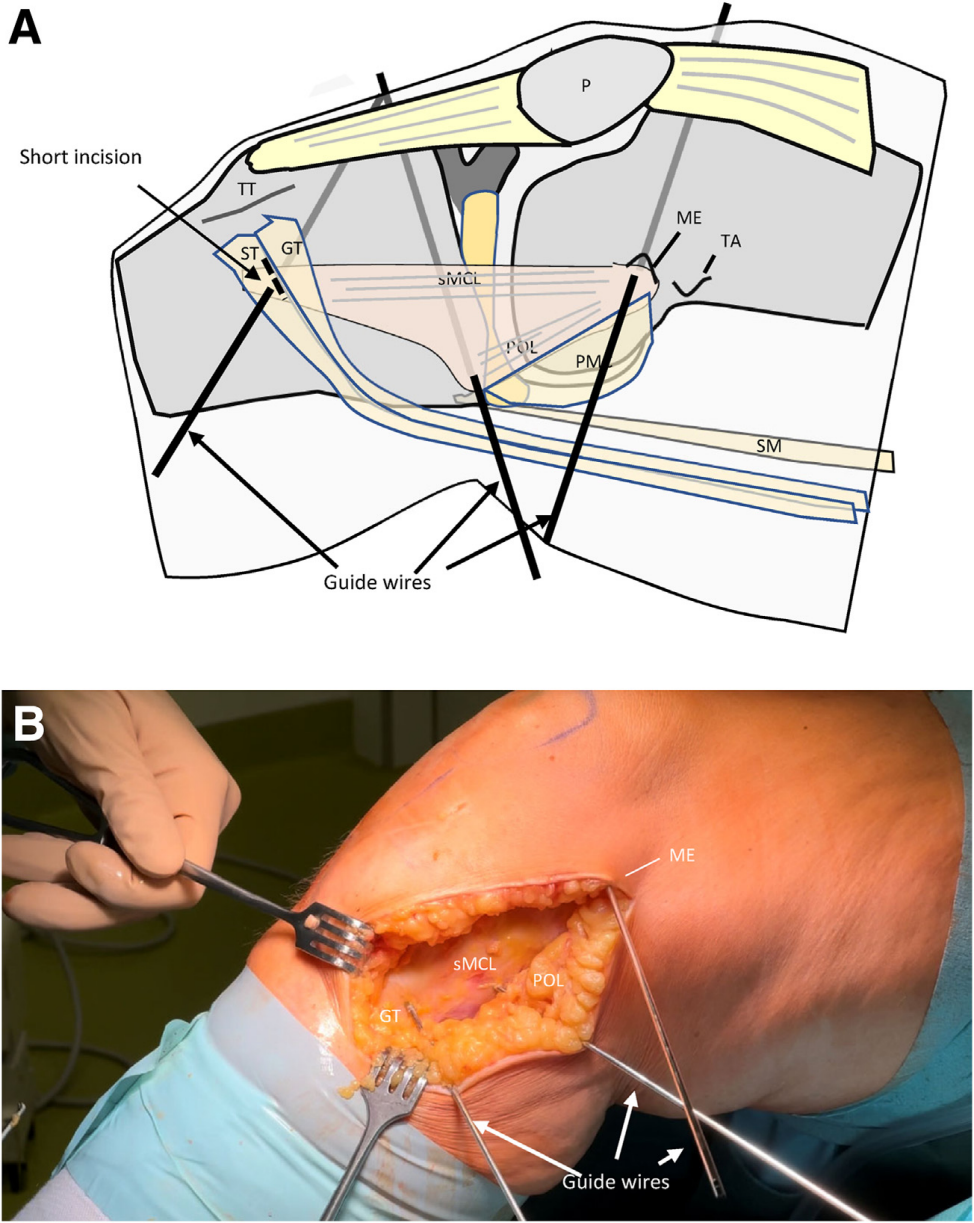
Drawing (a) and intraoperative photo (b) showing converging guidewires at the tibial insertions of sMCL and POL. Right knee of a patient in supine position. (AT, adductor tubercle; G, gracilis tendon; ME, medial epicondyle; P, patella; POL, posterior oblique ligament; PMC, posteromedial capsule; SE, semimembranosus tendon; sMCL, superior medial collateral ligament; ST, semitendinosus tendon, TT, tibial tuberosity.). Right knee of a patient in supine position.

After exposure of the medial collateral ligament complex, 2 guidewires are placed at the tibial insertions of the sMCL and POL and a third guidewire is placed between the femoral sMCL and POL insertion (Fig 5). The tibial sMCL insertion is located approximately 6 to 7 cm below the joint space in the middle of the tibial metaphysis covered by the hamstring tendons. To place the guidewire, a short incision is made between the semitendinosus and gracilis tendons. The tibial POL insertion is located proximal to the insertion of the semimembranosus tendon. The landmark for the femoral sMCL insertion is the medial epicondyle.^7^ The femoral insertion of the POL is 11 mm posterior to the medial epicondyle.^7^ Therefore, the common femoral tunnel for the sMCL and POL bundle is created just behind the medial epicondyle.

**Fig 6:**
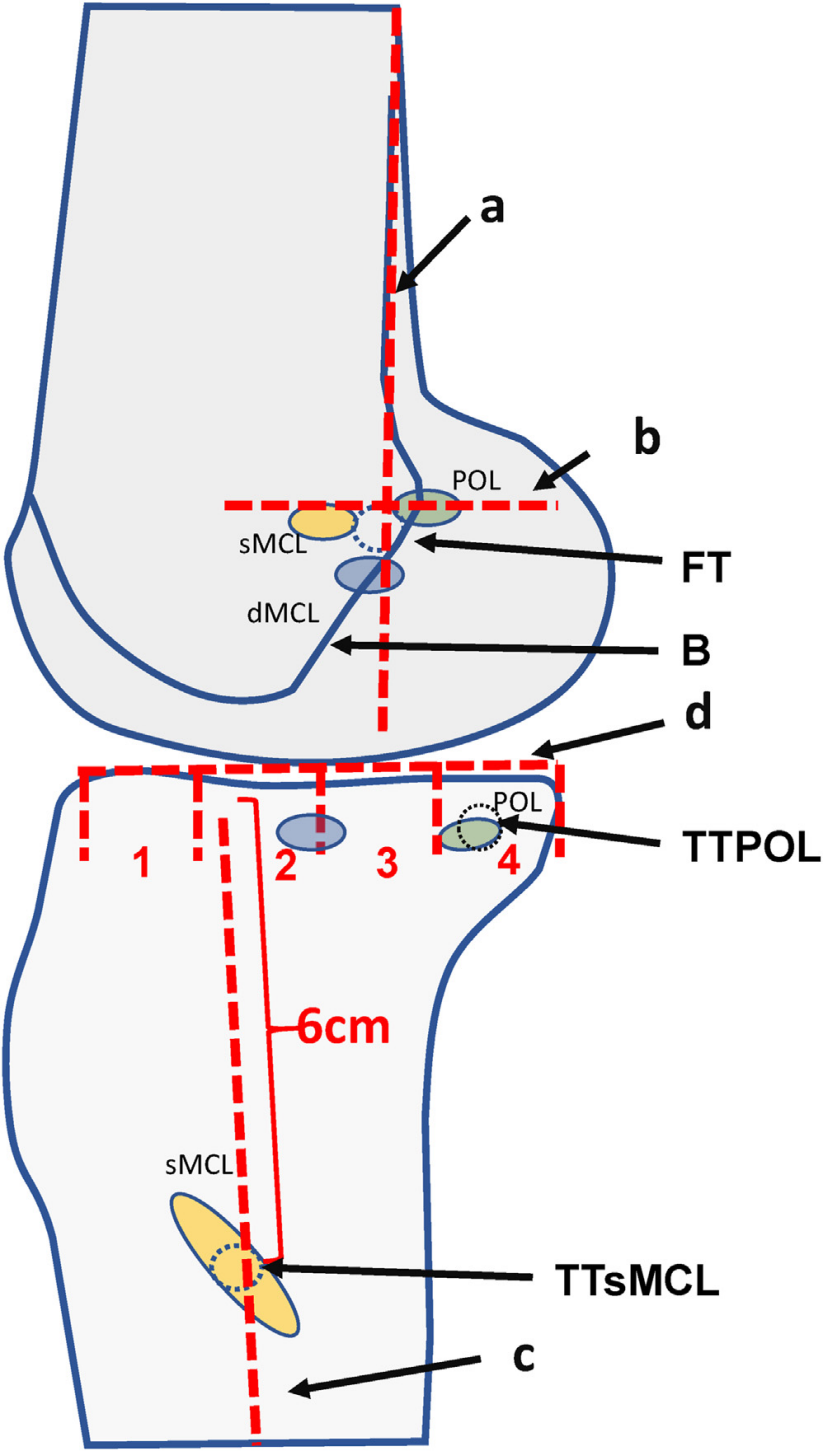
The drawing shows a projection of a radiographic lateral view of the knee joint, in which the position of the insertions of superior medial collateral ligament (sMCL; orange), deep medial collateral ligament (dMCL; blue), and posterior oblique ligament (POL; green) are marked according to Athwal et al.^13^ The common femoral tunnel (FT) lies on the extension of the posterior femoral cortex (a) just below a perpendicular line (b) that passes through the end of the Blumensaat line (B). The tibial sMCL tunnel (TTsMCL) lies on the bisecting line (c) of the proximal tibia approximately 6 to 7 cm below the tibial joint line (d). The tibial POL tunnel (TTPOL) lies in the posterior quadrant (4) about 1 cm below the tibial joint line (d). 1, 2, 3, 4 quadrants below the tibial joint line.

**Fig 7:**
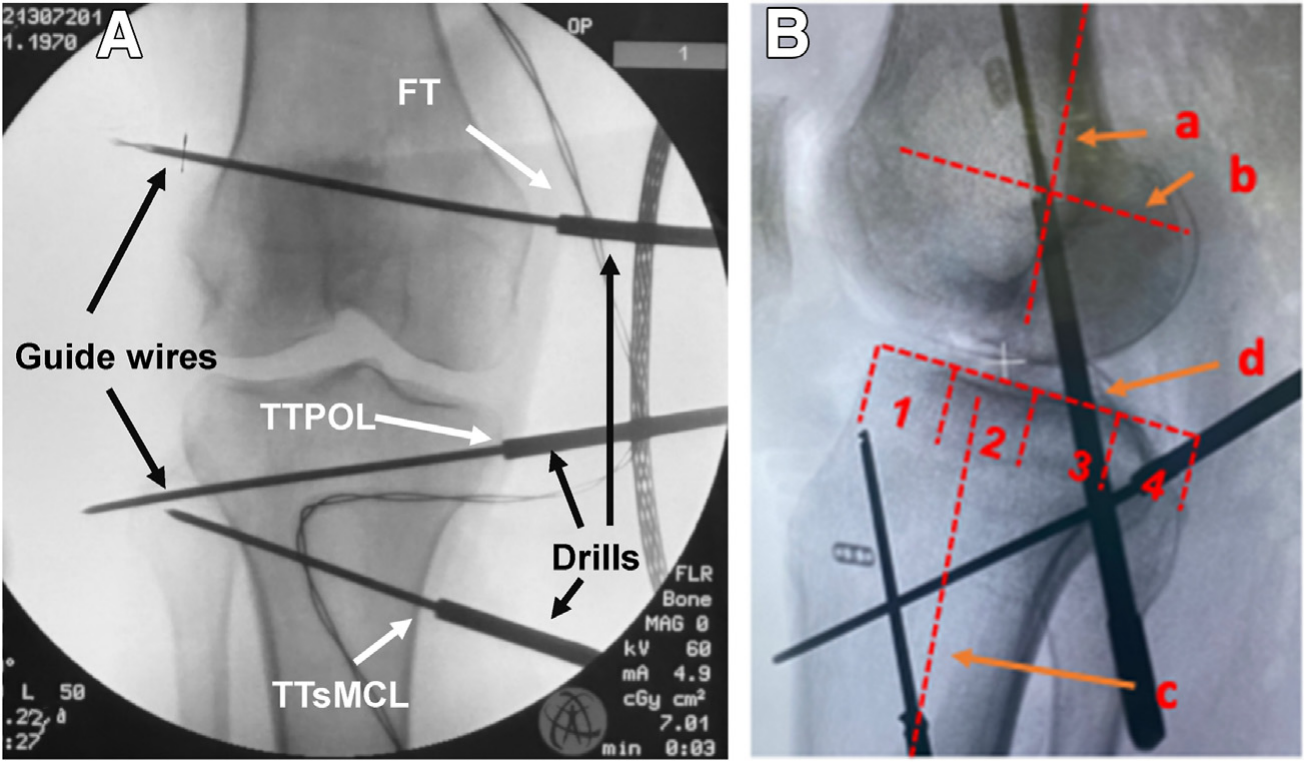
Anteroposterior (a) and lateral (b) fluoroscopic images to control the guidewire position. Drills advanced onto the cortex mark the entry of the guidewires into the bone. a: Extension of the posterior femoral cortex, b: perpendicular to a line passing through the end of the intercondylar line, c bisector of the tibia, d: joint line, 1, 2, 3, 4 quadrants below the tibial joint line. (FT, femoral tunnel; POL, posterior oblique ligament; sMCL, superior medial collateral ligament; TTPOL, tibial POL tunnel; TTsMCL, tibial sMCL tunnel.)

A fluoroscope is used to control the guidewire positions in 2 planes (Figs 6 and 7). In order to mark the entry of the guidewire into the cortical bone in the lateral plane, cannulated drills are advanced down to the bone. The position of the insertions of the sMCL and POL in the lateral plane is determined according to Athwal et al.^13^ (Fig 6).

**Fig 8:**
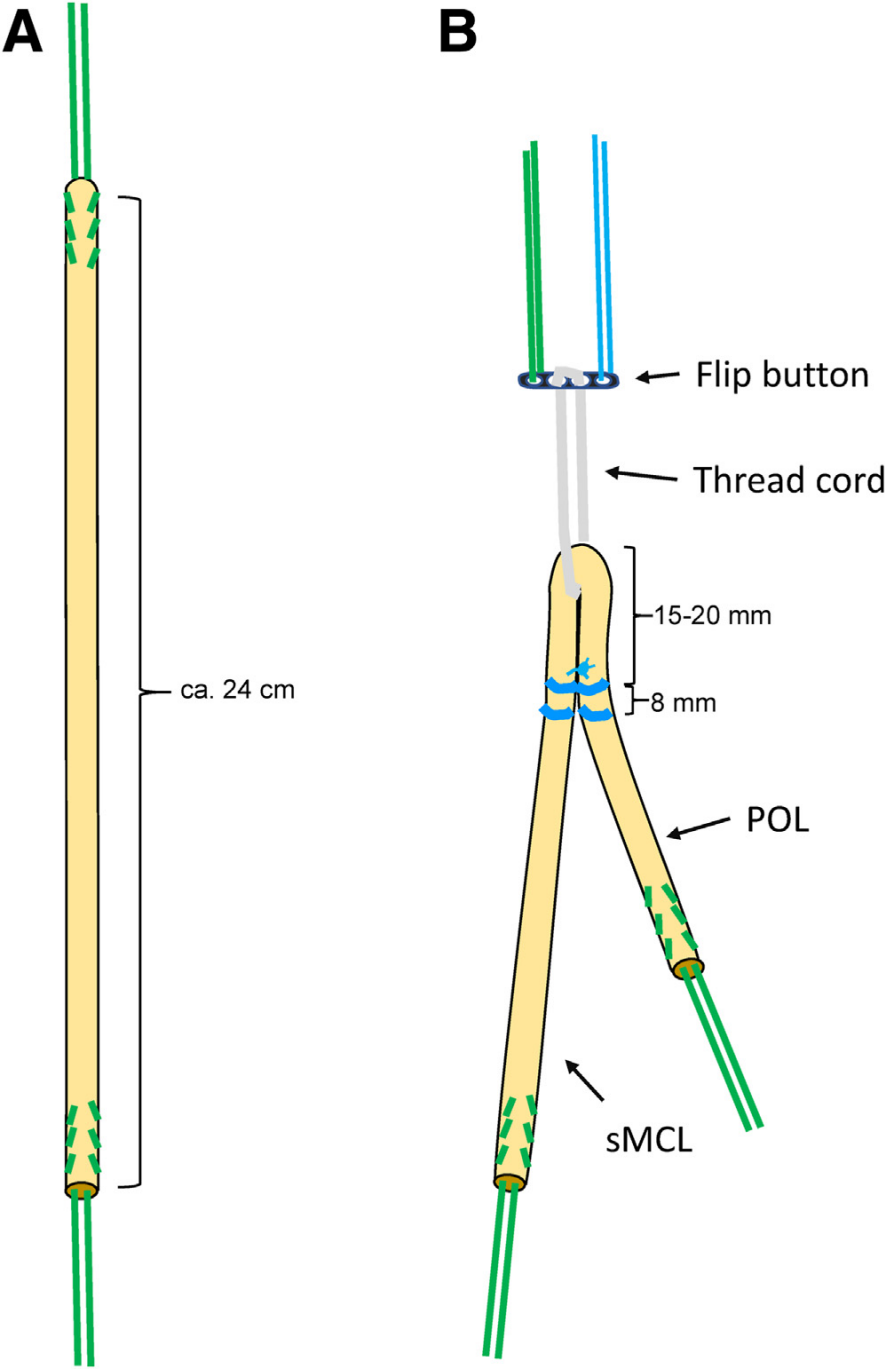
Tendon preparation. (a) Shortening of the semitendinosus tendon to a length of approximately 24 to 26 cm and reinforcement of both ends with a strong thread cord (e.g., FiberWire USP size 5; Arthrex). (b) Preparation of a graft consisting of 2 strands (longer strand: sMCL, shorter strand: POL). The graft loop is connected to a flip button using appropriate thread material. (POL, posterior oblique ligament; sMCL, superior medial collateral ligament.)

**Fig 9:**
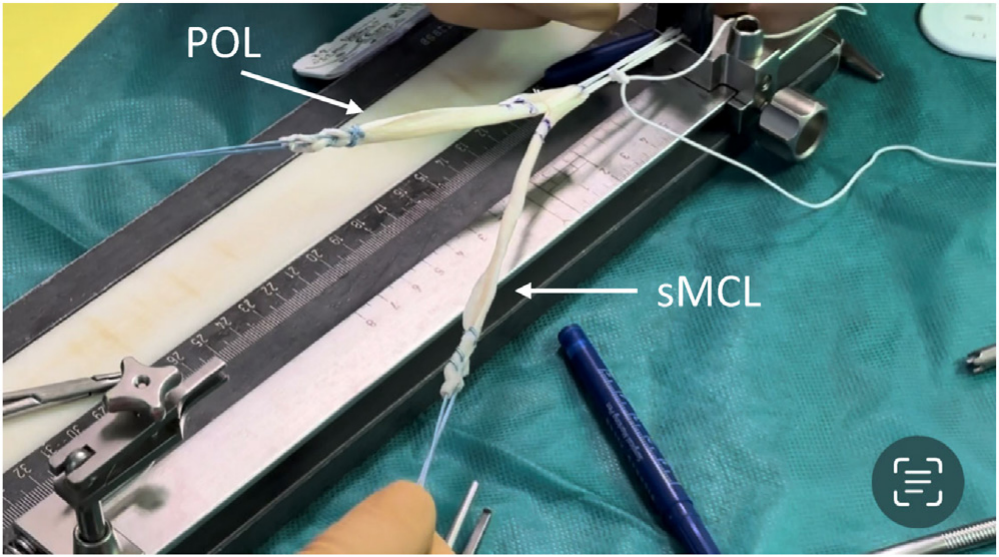
Photo of the 2-stranded graft. (POL, posterior oblique ligament; sMCL, superior medial collateral ligament.)

After guidewire placement, the tendon graft is prepared (Figs 8 and 9). First, the graft is shortened to a length of approximately 24 to 26 cm according to the distances measured between the guidewires. Both ends of the graft are reinforced with strong thread cords (e.g., FiberWire USP No. 5; Arthrex, Naples, FL). Then the graft is folded to a double-bundle graft. The loop of the graft is connected to an anchor (e.g., FLIPPTACK; Karl Storz, Tuttlingen, Germany) with a strong thread cord (e.g., FiberWire USP No. 5; Arthrex). Alternatively, freely adjustable buttons (e.g., ACL TightRope, Arthrex) or continuous-loop buttons (e.g., EndobuttonCL, Smith & Nephew, London, UK) also can be used. After preparation, the graft is placed in a vancomycin solution for approximately 5 minutes (1 mg/mL).^14^

**Fig 10:**
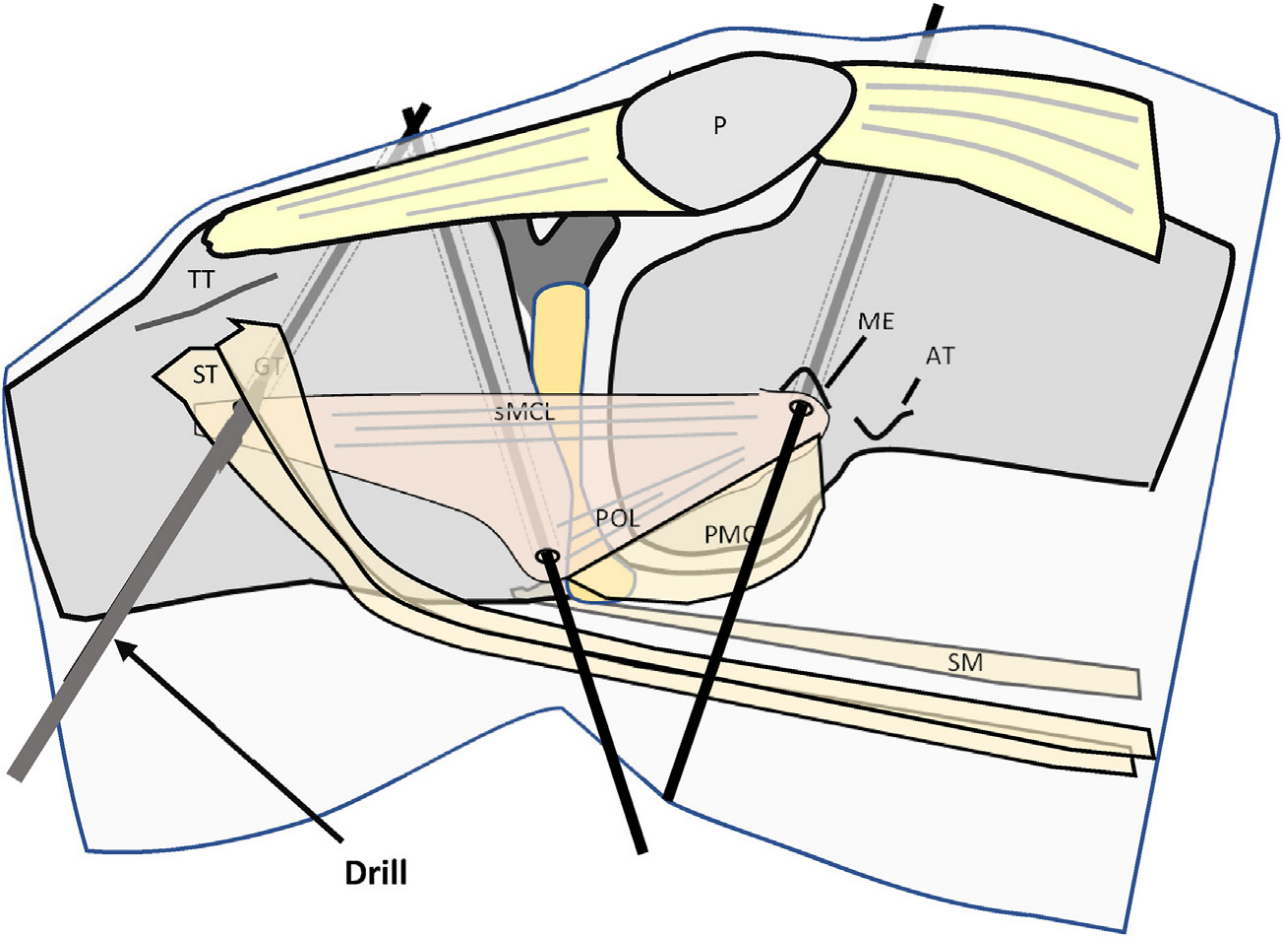
Overdrilling of the 3 guidewires with a drill (diameter 4.5 mm) up to the lateral cortex of the femur and tibia. Right knee of a patient in supine position. (AT, adductor tubercle; G, gracilis tendon; ME, medial epicondyle; P, patella; SM, semimembranosus tendon; ST, semitendinosus tendon.)

**Fig 11:**
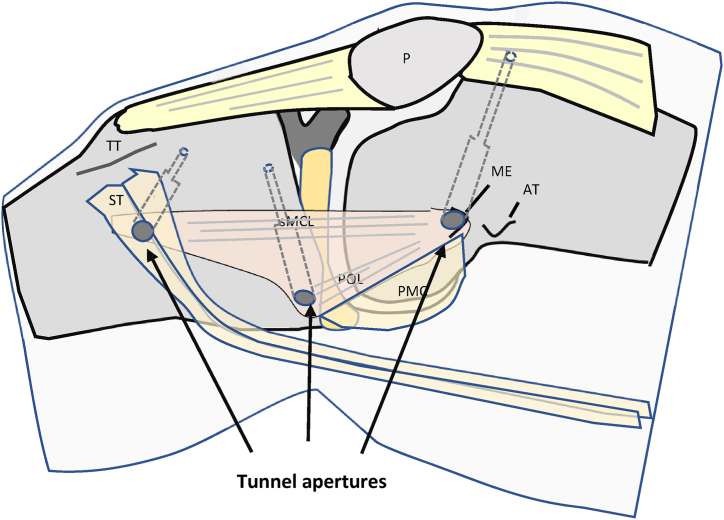
Blind tunnels are drilled to a length of approximately 30 mm with a drill with a larger diameter depending on the diameter of the graft. Right knee of a patient in supine position.

**Fig 12:**
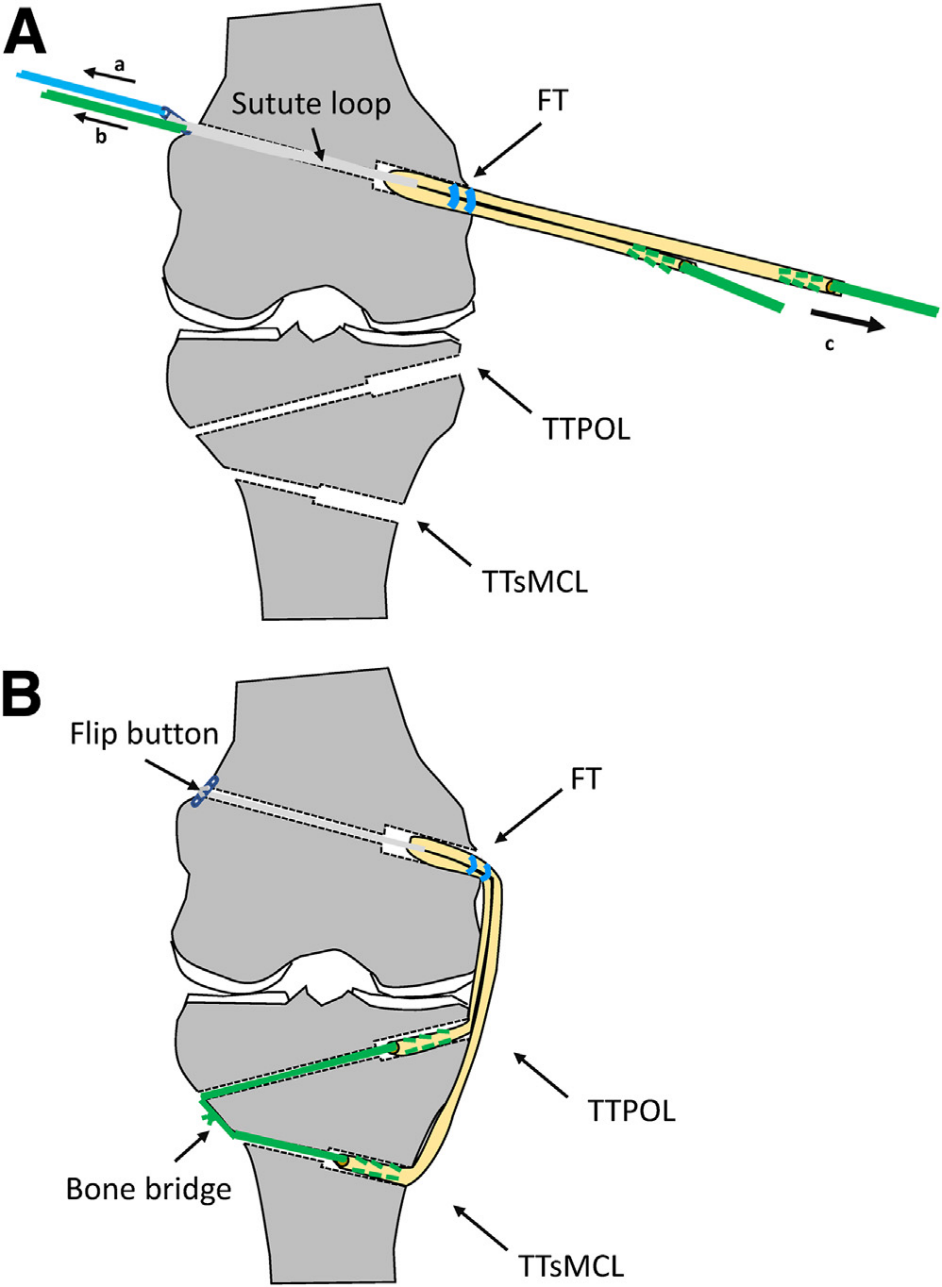
Pulling the graft into the bone tunnels. a) First, the graft loop is pulled into the femoral bone tunnel by using an eyelet wire with a suture loop (a) and extracortical fixation is performed with a button (b). After successful fixation cyclic traction is applied (c) to the graft to seat the anchor on the femoral cortex and condition the graft construct. b) Pulling the POL and the sMCL strand into the corresponding tibial bone tunnels and cyclic movement of the knee joint while pulling on both graft strands. Then the threads of the graft ends are tied over the lateral cortical bone bridge in approximately 20° flexion.

After graft preparation, a cannulated 4.5-mm drill is used to drill the 3 tunnels. Then, blind tunnels are drilled to a length of approximately 30 mm with another drill with a larger diameter according to the diameter of the graft (Figs 10 and 11). After the tunnels are complete, the graft loop is pulled into the femoral bone tunnel by using an eyelet wire with a suture loop. Extracortical fixation is performed with a button (Fig 12). After successful fixation, cyclic traction is applied to the graft to condition the construct.

**Fig 13:**
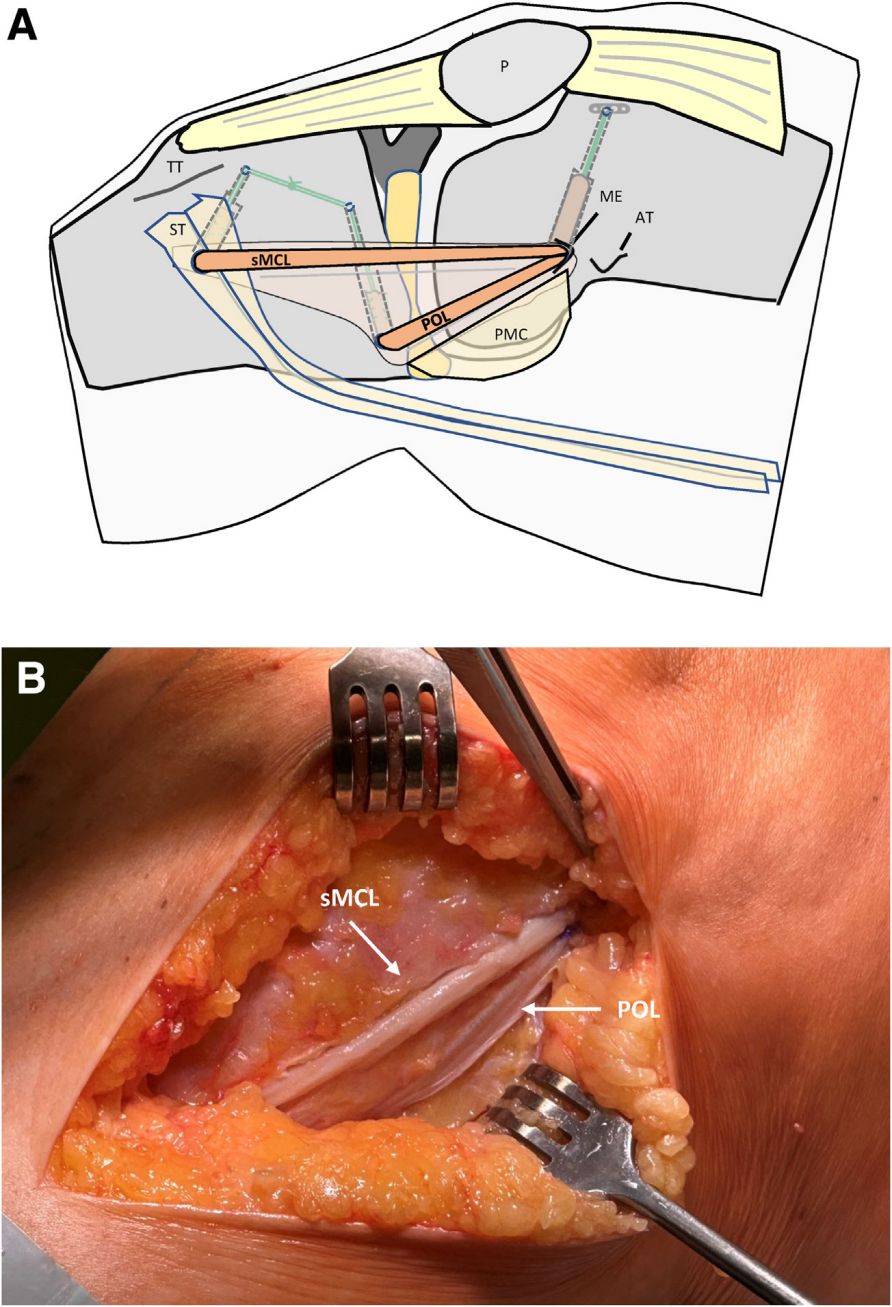
This drawing (a) and intraoperative photo (b) show the 2 tubular graft strands for sMCL and POL after femoral and tibial fixation. Right knee of a patient in supine position. (AT, adductor tubercle; G, gracilis tendon; ME, medial epicondyle; P, patella; POL, posterior oblique ligament strand; SE, semimembranosus tendon; sMCL, superior medial collateral ligament strand; PMC, posteromedial capsule; ST, semitendinosus tendon; TT, tibial tuberosity.)

**Fig 14:**
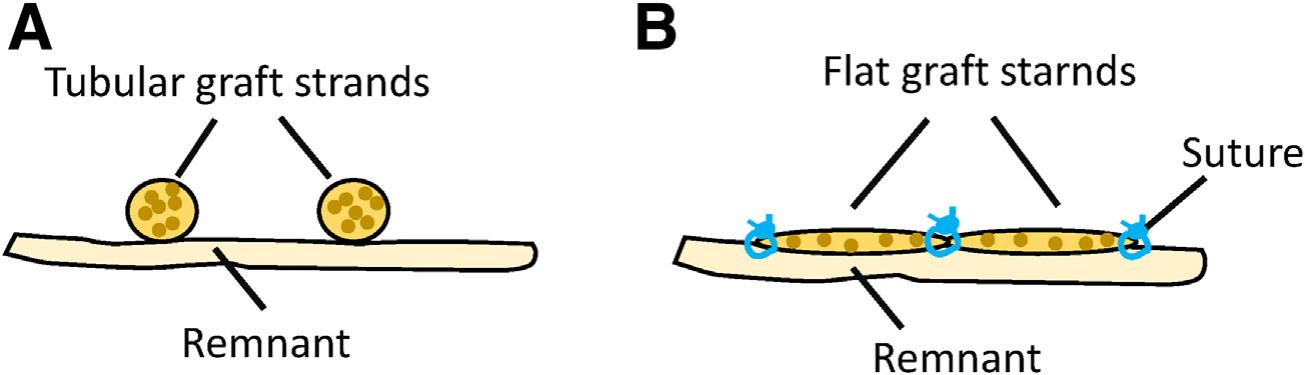
Schematic drawing showing tubular and flat grafts. (a) Tubular graft strands. (b) The peripheries of both graft strands are connected to each other and to the residual tissue with absorbable sutures (e.g., polydioxanone, gauge 2-0). Thus, the tubular grafts are pulled apart to form a flat shape.

**Fig 15:**
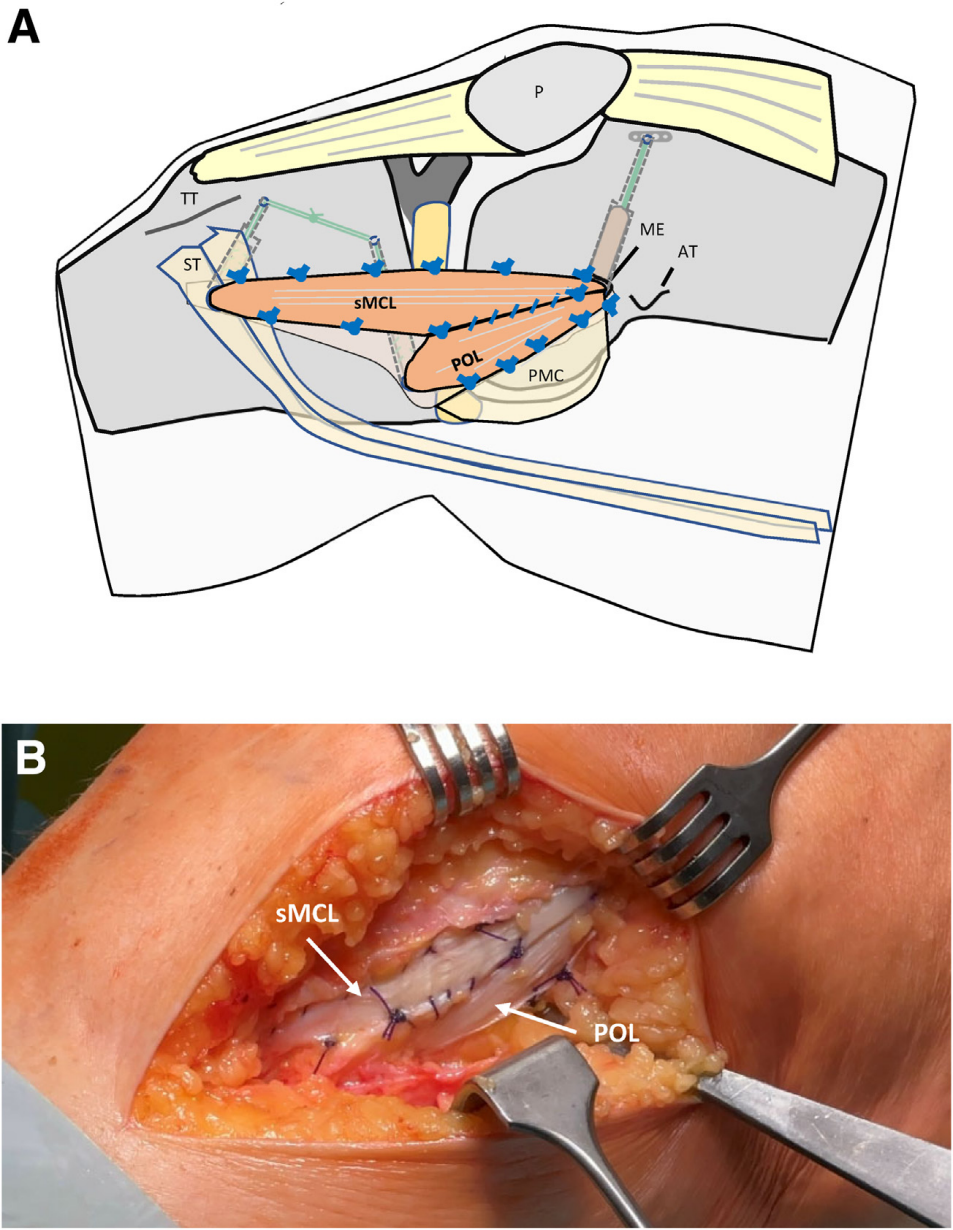
This drawing (a) and intraoperative photo (b) show the 2 flat graft strands after suturing to each other and to the residual tissue of the posteromedial complex. Right knee of a patient in supine position. (AT, adductor tubercle; G, gracilis tendon; ME, medial epicondyle; P, patella; POL, posterior oblique ligament; SE, semimembranosus tendon; sMCL, superior medial collateral ligament; ST, semitendinosus tendon; TT, tibial tuberosity.)

Then, the POL and the sMCL strands are pulled into the corresponding tibial bone tunnels. After the knee joint is cycled several times while pulling on both graft strands with maximal manual force, the reinforcement threads are tied over the lateral cortical bone bridge in approximately 20° flexion (Figs 12 and 13). After fixation, the peripheries of both graft strands are connected to each other and to the residual tissue with absorbable sutures (e.g., polydioxanone, gauge 2-0). Thus, the tubular grafts are pulled apart to form a flat shape (Figs 14 and 15).

Postoperatively, the patient is mobilized with partial weight-bearing (approximately 10 kg) on the operated leg for 6 weeks. A movable knee brace (e.g., Genu Arexa; Otto Bock Health Care, Duderstadt, Germany) is applied for 8 weeks (movement 0-5-90° for 2 weeks and 0-0-120° for another 4 weeks, then free).

## Discussion

In recent expert consensus statements, there was agreement that for chronic posteromedial laxity, anatomic reconstruction of the sMCL and POL is recommended.^5^^,^^6^ In 2009, Lind presented a 2-bundle technique for the reconstruction of both structures.^8^ The ipsilateral semitendinosus tendon is detached from the muscle, the tibial insertion remains intact, the tendon loop is anchored in a femoral tunnel at the medial epicondyle and the free end is anchored as a POL graft below the posterior tibial plateau.

Lind et al.^8^ reported in their retrospective case series approximately 98% normal or nearly normal medial stability (International Knee Documentation Committee grade A or B). This technique can therefore be considered the gold standard and most widely accepted medial reconstruction technique today.

Due to the reconstruction of the 2 biomechanically important structures sMCL and POL, this technique is commonly referred to as an anatomical technique.^4^ However, 2 important anatomical features of the posteromedial complex are not considered here. First, the semitendinosus insertion is more anterior than the distal sMCL insertion, and second, the ligamentous structures of the posteromedial complex do not have a round tubular structure but are flat in shape.^1^^,^^2^^,^^7^^,^^15^^,^^16^ Another functional disadvantage of this technique is the use of the ipsilateral semitendinosus tendon, since the medial flexors also make an important contribution to stabilization against valgus stress.^17^

In the technique presented here, an allograft or free tendon graft of the contralateral side is used to spare the ipsilateral flexors. However, care was also taken to ensure that the surgical technique is not too complicated from a surgical point of view (Table 4). With the tibial bone tunnels, care is taken to ensure that these are in the natural insertion area of the sMCL and POL. Due to the anatomical proximity of the sMCL and POL insertion, a common tunnel is created femorally. A biomechanical study has shown that this 2-bundle technique can restore both valgus and posterior stability.^9^

**Table 4:** Complications and Prevention

Complication	Prevention
Tunnel malplacement	Control guidewire position with the fluoroscope
Infection	Soaking the graft in vancomycin solution (1 mg/mL), preoperative antibiotics (eg, cephazolin 1 g)
Postoperative deficit in range of motion	Anatomic tunnel locations, postoperative physiotherapy with passive and active exercises

From our point of view, the use of a flat graft also has several advantages. Due to the wider arrangement of the fibers, the normal tension behaviour is more likely to be imitated and the ligamentization of the graft seems to be improved, since there is more contact with the remnants of the posteromedial complex. We believe that by suturing the graft onto the remnants, the graft integrates into the existing structures of the posteromedial complex, optimizing their biomechanical function as well. Further biomechanical and animal studies are needed to prove this hypothesis.

## Conflict of interest

The authors report the following potential conflicts of interest or sources of funding: W.P. reports consulting activity for Karl Storz and OPED and lecture activity for Otto Bock Healthcare, Plasmaconcept, and Geistlich. All other authors (H.A.M., J.B., M.H., K.B.) declare that they have no known competing financial interests or personal relationships that could have appeared to influence the work reported in this paper. Full ICMJE author disclosure forms are available for this article online, as supplementary material.

## Supplementary Data

Video 1 This video shows the technique of reconstruction of the medial collateral ligament complex with a flat allograft semitendinosus tendon.
